# CREATING CONNECTIONS BETWEEN PEOPLE LIVING WITH DEMENTIA AND VOLUNTEERS THROUGH ART

**DOI:** 10.1093/geroni/igae098.1508

**Published:** 2024-12-31

**Authors:** Meghan Young

**Affiliations:** Miami University, Oxford, Ohio, United States

## Abstract

Scripps Gerontology Center’s award-winning Opening Minds through Art (OMA) program has built bridges across age and cognitive barriers through art for 17 years. People living with dementia, also known as artists, and volunteers are paired 1:1 for weekly abstract art making sessions. The program philosophy is based on autonomy and the freedom to make choices within a structured process, so the artists have the opportunity to fully immerse themselves in art-making with the assistance of their volunteer partners. Volunteers in the program are trained to rely on the imagination and skills of the artist, without making any artistic, aesthetic decisions during the art-making process. Over the course of the art-making sessions, relationships develop between the pairs. Thirteen published studies show the impact of the program including findings to support that volunteers are more positive in their attitudes toward people living with dementia post-participation, and participants living with dementia displayed more interest and pleasure during OMA than during traditional visual art activities. This session will provide an overview of the program and a description of previous research as background information for the remainder of the symposium. The session will also include an overview of OMA’s first and longest running program at Miami University where 2,898 older adults and 2,812 students have participated in OMA through in-person and virtual intergenerational programming. To highlight OMA’s longevity, we will also present an original compilation of internal program evaluation data to map the expansion and growth of OMA from inception to the present day.

